# Stage-Dependent Heat-Stress Mechanisms in Chinese Sturgeon (*Acipenser sinensis*) Early Life-Stages Under Simulated Thermal-Lag Effect

**DOI:** 10.3390/biology15191713

**Published:** 2026-09-26

**Authors:** Xiaoqian Leng, Junlin Yang, Xinchi Li, Qiong Zhou, Ke Zhang, Jiang Luo, Wei Xiong, Jinping Wu, Luoxin Li, Yuan Liu, Jinming Wu, Junyi Li

**Affiliations:** 1 Yangtze River Fisheries Research Institute, Chinese Academy of Fishery Sciences, Wuhan 430223, China; 2 College of Fisheries and Life Science, Shanghai Ocean University, Shanghai 201306, China

**Keywords:** Chinese sturgeon, thermal lag effect, early-life-stage development, proteomics, metabolomics

## Abstract

Hydraulic engineering-driven thermal lag disturbs natural water temperature and induces thermal stress in early-life-stage Chinese sturgeon (*Acipenser sinensis*), yet stage-specific molecular stress mechanisms remain unclear. This study assessed phenotypic, proteomic and metabolomic responses of endogenous-nutrition embryos and exogenous first-feeding larvae under 16 °C, 19 °C and 22 °C. Phenotypic results showed optimal embryo survival at 16 °C (hatching rate 64.6%), maximum larval growth at 19 °C (specific growth rate 6.79%/d), and severe developmental suppression at 22 °C (with the hatching rate dropping by 49.6 percentage points relative to 16 °C). Multi-omics uncovered distinct injury pathways: high temperature disrupted amino-acid and fatty-acid catabolism and triggered organic-acid accumulation and energy deficiency in embryos; in first-feeding larvae, it activated ferroptosis, polyunsaturated fatty-acid peroxidation and lysosomal pathways to produce oxidative damage. These findings demonstrate that a 1–3 °C temperature rise exceeds stage-specific thermal tolerance, with embryos succumbing to “metabolic suppression–energy deficiency” and larvae to “oxidative stress–ferroptosis”. This study provides the first integrative proteomic and metabolomic evidence of stage-dependent thermal injury mechanisms in sturgeon, offering mechanistic insights critical for conservation physiology and informing conservation actions for this endangered sturgeon species.

## Simple Summary

Chinese sturgeon is an endangered fish species that lives in the Yangtze River in China. Dams built on the river have changed the natural water temperatures during the sturgeon’s breeding season, making the water warmer than it used to be. This study investigated how increased temperature harms sturgeon in two early life phases: developing embryos that live off internal yolk reserves, and newly feeding young fish that start eating external food. We kept sturgeon at three steady temperatures and recorded survival and growth, together with broad-scale molecular measurements. Low-temperature conditions favored embryo survival; a moderate temperature supported larval growth, while the highest temperature greatly reduced hatching and survival. Warming damaged embryos mainly by disturbing nutrient use and causing energy shortages. By contrast, in newly feeding young fish, heat triggered oxidative harm and iron-related programmed cell death. Our findings show that even small temperature rises exceed stage-specific thermal limits. This knowledge supports practical conservation work to protect natural reproduction of this rare river fish.

## 1. Introduction

The Chinese sturgeon (*Acipenser sinensis*), a rare and endangered fish endemic to China, serves as a flagship species and a vital indicator organism of the Yangtze River aquatic ecosystem. Affected by the superimposed impacts of damming, shipping, and climate change, its natural population has undergone a sustained decline [1,2,3] and it is now listed as Critically Endangered (CR) by the International Union for Conservation of Nature (IUCN). Despite long-term implementation of artificial propagation and release, natural reproduction events have not been detected for many consecutive years at the only known natural spawning ground downstream of the Gezhouba Dam [4], underscoring the urgent need to elucidate the underlying driving environmental mechanisms.

Water temperature is a critical environmental factor governing the success of early fish life history, directly influencing embryonic development rate, hatching efficiency, and larval survival and growth [5,6,7]. The natural spawning season of Chinese sturgeon occurs from October to December each year, when the water temperature of the Yangtze River exhibits a natural declining trend, and embryos and larvae complete their early development under relatively low-temperature conditions. However, the “thermal lag effect” induced by the operation of hydraulic engineering has caused the water temperature in spawning grounds to rise by 1–3 °C compared to historical levels and has altered the natural thermal regime of the water body [8,9]. In recent years, continuous monitoring further indicates that the warming trend of the Gezhouba-downstream spawning habitat has persisted, with autumn water temperatures frequently exceeding 21 °C [10]. For Chinese sturgeon, with a natural reproductive thermal window of 16–20 °C, the abnormal increase in water temperature during the spawning period may constitute a significant ecological stress [11].

Existing studies have shown that temperature deviation from the optimal range significantly reduces fertilization and hatching rates and increases the deformity rate in fish; however, the underlying molecular mechanisms, particularly the stage-specific responses to temperature during different embryonic developmental phases, remain poorly understood [12,13]. During the early life history of fish, the embryonic incubation stage and the larval first-feeding stage differ fundamentally in metabolic mode, energy source, and environmental sensitivity [14,15,16]. Embryos at the incubation stage rely entirely on endogenous yolk reserves for anaerobic metabolism, whereas larvae at the first-feeding stage switch to an aerobic exogenous metabolic mode. This transition causes an abrupt increase in reactive oxygen species (ROS), which may alter the response thresholds of oxidative stress-related pathways. However, most traditional studies have treated embryos and larvae as a whole, overlooking such stage-specific thermal requirements and the associated differences in molecular regulation [17]. Furthermore, existing reports have largely focused on phenotypic observations, lacking systematic integrative analysis from the proteome to the metabolome. In particular, no definitive conclusions have been reached on core scientific questions such as through which pathways high temperature inhibits development and whether response mechanisms shift between different developmental stages [18].

To address the aforementioned research gaps, this study, for the first time, integrated proteomics and metabolomics to systematically compare the molecular response profiles of Chinese sturgeon at the embryonic incubation stage (endogenous nutrition stage) and the larval first-feeding stage (exogenous feeding stage) under three temperature regimes: 16 °C (historical suitable spawning temperature), 19 °C (intermediate warming scenario), and 22 °C (elevated extreme thermal condition recorded in spawning habitats). This study aimed to test the following hypotheses: (1) the two critical stages of early Chinese sturgeon development possess significantly differentiated thermal tolerance windows and molecular response patterns; (2) at the incubation stage, heat stress mainly disrupts the programmed catabolism of endogenous amino acids and fatty acids in the yolk, triggering a cascade of “metabolic inhibition–energy deficiency–structural and functional damage”; whereas at the first-feeding stage, due to the predominance of aerobic metabolism, the stress mechanism shifts to a damage pathway driven by “oxidative stress–lipid peroxidation–ferroptosis/lysosomal decompensation”. By elucidating stage-specific molecular mechanisms of thermal stress across two critical early ontogenetic phases, this study provides mechanistic insights into how thermal-lag warming impairs early-life development of endangered *Acipenser sinensis*, advancing our knowledge in fish conservation physiology.

## 2. Materials and Methods

### 2.1. Experimental Design and Sample Collection

The fertilized eggs of Chinese sturgeon used in this study were obtained via artificial propagation. One female and three male broodfish with favorable gonadal development status were selected for hormonal induction and artificial insemination [19] so as to broaden the genetic background of the experimental offspring. The fertilized eggs were placed in mesh-screen hatching boxes (60 cm × 50 cm × 20 cm), which were floated in a circular fiberglass tank (diameter 2 m, height 0.5 m) for incubation. Three constant-temperature treatments were established: 16 °C, 19 °C, and 22 °C groups, with the temperature maintained using heaters (±0.5 °C). Each treatment consisted of three replicate hatching boxes, and 500 fertilized eggs were placed in each box. Micro-flow water and continuous aeration were supplied during incubation, and water temperature was monitored in real time.

The fertilized eggs in each temperature group were continuously observed. The criterion for determining developmental stages was the time when 50% of individuals exhibited characteristic features, and the time point of each developmental stage was recorded. The numbers of dead embryos, gastrula-stage embryos, hatched larvae, and surviving larvae at the first-feeding stage were counted. The total length and body weight of newly hatched larvae, yolk-sac larvae, and larvae at 3 days after first feeding were measured. For omics sequencing, six biological replicates (*n* = 6) were collected per temperature group at each stage: newly hatched larvae (incubation stage) and first-feeding larvae (exogenous feeding stage). Each biological replicate consisted of a pooled sample of multiple individuals, evenly sampled from the three replicate hatching boxes to avoid box-specific effects. All samples were snap-frozen in liquid nitrogen and stored at −80 °C.

### 2.2. Protein Identification and Quantification

Frozen samples stored at −80 °C were ground to a fine powder in liquid nitrogen. An appropriate amount of powder was transferred to a 1.5 mL centrifuge tube, and lysis buffer (containing 8 mol/L urea, 1 mmol/L PMSF, and 2 mmol/L EDTA) was added. The mixture was sonicated on ice for 5 min and centrifuged at 15,000 g for 10 min at 4 °C. The supernatant was collected, and the protein concentration was determined using a BCA assay. A 100 μg aliquot of protein was diluted to 200 μL with 8 mol/L urea, reduced with DTT (final concentration 5 mmol/L) at 37 °C for 45 min, and alkylated with iodoacetamide (final concentration 11 mmol/L) at room temperature in the dark for 15 min. Subsequently, 800 μL of 25 mmol/L ammonium bicarbonate and 2 μL of trypsin were added, and digestion was performed overnight at 37 °C. After digestion, the pH was adjusted to 2–3 with 20% trifluoroacetic acid. Peptides were desalted using C18 solid-phase extraction, and the peptide concentration was measured with a quantitative peptide assay kit (Thermo Fisher Scientific, Waltham, MA, USA).

Peptide separation was performed on a Vanquish Neo UHPLC system (Thermo Fisher Scientific, Bremen, Germany) with mobile phase A (0.1% formic acid in water) and mobile phase B (0.1% formic acid in acetonitrile), using a PepMap Neo Trap Cartridge (Thermo Fisher Scientific, Bremen, Germany) as the trapping column and an Easy-Spray™ PepMap™ Neo analytical column (Thermo Fisher Scientific, Bremen, Germany). The column temperature was maintained at 55 °C, with a loading amount of 200 ng, a flow rate of 2.5 μL/min, an effective gradient time of 6.9 min, and a total run time of 8 min. The separated peptides were analyzed on an Orbitrap Astral high-resolution mass spectrometer (Thermo Fisher Scientific, Bremen, Germany) in data-independent acquisition (DIA) positive-ion mode. The full scan range was 380–980 m/z with a resolution of 240,000, and 299 isolation windows (2 Th width) were set, with an HCD collision energy of 25%.

Raw mass spectrometric data were searched against the uniprotkb_taxonomy_id_7901_2026_02_15.fasta database (50,648 sequences) using DIA-NN software (v1.8.1) in library-free mode. Deep learning-based spectral library prediction was enabled, and the match-between-runs (MBRs) function was activated to generate a spectral library from the DIA data for subsequent re-analysis. Protein and precursor identifications were filtered at a false discovery rate (FDR) < 1%. Protein quantification was performed using the MaxLFQ algorithm, then the intensity values of each protein across samples were extracted and normalized by the sample-wise median to obtain relative quantitative values. Differentially expressed proteins were identified using fold change and statistical tests: for two-group comparisons, the ratio of the mean quantitative values of biological replicates was used as the fold change, and a *t*-test was applied to calculate *p*-values; for multi-group comparisons, ANOVA was performed. *p*-values were corrected using the Benjamini–Hochberg method to obtain FDR.

### 2.3. Widely Targeted Metabolomic Identification and Quantification

Samples stored at −80 °C were thawed on ice and ground thoroughly in liquid nitrogen. A 20 mg portion of the ground sample was mixed with 400 μL of extraction solvent (methanol:water = 7:3, *v*/*v*) containing internal standards, vortexed at 1500 rpm for 5 min, and left to stand on ice for 15 min. After centrifugation at 12,000 rpm for 10 min at 4 °C, 300 μL of the supernatant was collected, kept at −20 °C for 30 min, and centrifuged again at 12,000 rpm for 3 min at 4 °C. A 200 μL aliquot of the supernatant was used for LC-MS analysis.

All samples were analyzed in both positive- and negative-ion modes. Chromatographic separation was performed on a Waters UPLC Premier HSS T3 column (1.8 μm, 2.1 mm × 100 mm; Waters Corporation, Milford, MA, USA). Mobile phase A was 0.1% formic acid in water, and mobile phase B was 0.1% formic acid in acetonitrile. The elution gradient was as follows: 0–2 min, B increased from 5% to 20%; 2–5 min, to 60%; 5–6 min, to 99% and held for 1.5 min; then returned to 5% within 6.1 min and equilibrated for 2.4 min. The column temperature was 40 °C, the flow rate was 0.4 mL/min, and the injection volume was 3 μL. Mass spectrometric detection was carried out on a QE mass spectrometer (Thermo Fisher Scientific, Bremen, Germany) with electrospray ionization. Full scan was performed over the m/z range of 70–1000 at a resolution of 60,000. The spray voltage was 3.8 kV in positive mode and 3.4 kV in negative mode; the sheath gas flow rate was 60 Arb, and the auxiliary gas flow rate was 20 Arb. The ion transfer tube temperature was set at 320 °C and the vaporizer temperature at 350 °C. Collision energies of 30, 40, and 50 V were applied. Data-dependent acquisition (Top 10) was used with dynamic exclusion enabled.

Raw data files were converted to mzML format using ProteoWizard and processed with XCMS for peak detection, alignment, and retention time correction. Peaks with a missing rate >50% across samples were filtered out. Missing values were imputed using the KNN method (missing rate <50%) or replaced with 1/5 of the minimum value (missing rate >50%), and peak areas were corrected by the SVR method. Metabolite identification was performed by searching an in-house database integrated with public and predicted libraries. Metabolites with a composite score >0.5 and a QC sample coefficient of variation <0.3 were retained. Data from positive- and negative-ion modes were merged and deduplicated to produce the final data matrix.

### 2.4. Bioinformatics Analysis

The principal component analysis (PCA) and hierarchical cluster analysis (HCA) were performed on both proteomic and metabolomic data. Differentially expressed proteins (DEPs) were screened using the mean ratio among biological replicates as fold change, with thresholds of FC ≥ 1.5 or FC ≤ 0.6667 and *p* ≤ 0.05. Differentially accumulated metabolites (DAMs) were selected using OPLS-DA VIP > 1 and *p* < 0.05 (log_2_-transformed data, 200-iteration permutation test). All differentially abundant molecules were subjected to KEGG annotation and pathway enrichment analysis.

## 3. Results

### 3.1. Phenotypic Differences in Chinese Sturgeon Embryos and Larvae Under Different Temperature Gradients

Under the three constant-temperature regimes of 16 °C, 19 °C, and 22 °C, phenotypic parameters, including fertilization rate, hatching duration, hatching rate, larval survival rate, and specific growth rate, all exhibited significant intergroup differences (*p* < 0.05) (Table 1).

At 16 °C, embryo survival-related traits were optimal: the fertilization rate was 97.5% ± 0.6%, the hatching rate was 64.6% ± 0.8%, and the survival rate of newly hatched larvae was 87.6% ± 2.3%; however, the embryonic development rate was the slowest, with a hatching duration of up to 170 h. At 19 °C, embryonic development was markedly accelerated, with hatching time shortened to 104 h, and larval specific growth rate reached the peak value across all temperature gradients (6.79% ± 0.93%/d), but the hatching rate dropped sharply to 23.1% ± 3.4%. At 22 °C, embryonic development was the fastest (hatching required only 84 h), yet reproductive and growth indicators deteriorated comprehensively: compared with the 16 °C group, the fertilization rate decreased by 2.9 percentage points, hatching rate by 49.6 percentage points, larval survival rate by 12.2 percentage points, and larval specific growth rate fell back to 5.95% ± 2.20%/d.

Pearson correlation analysis revealed that incubation temperature was very strongly negatively correlated with fertilization rate (r = −0.984), hatching rate (r = −0.932), larval survival rate (r = −0.964), and hatching duration (r = −0.955), whereas only a weak positive correlation was observed with larval specific growth rate (r = 0.703). Taken together, the phenotypic data indicate that 16 °C was most favorable for embryo survival, 19 °C better promoted rapid larval growth, and 22 °C significantly suppressed overall development.

### 3.2. Proteomic Differential Response Characteristics

PCA of the proteomic data showed that samples from the embryonic incubation stage and the first-feeding stage were clearly separated along the first principal component, while different temperature treatments within each stage exhibited a generally consistent gradient distribution along the second principal component (Figure 1a). This indicated that developmental stage was the primary source of variation in protein expression profiles, and that temperature effects shared a similar directionality across the two stages. A total of 931 temperature-responsive significantly differentially expressed proteins (DEPs) were identified (|log_2_FC| ≥ 0.5, *p* < 0.05, Table S1). Among them, 340 DEPs were detected at the incubation stage and 637 DEPs at the first-feeding stage (Figure 1b, Table S2), with 46 DEPs common to both stages (mainly heat shock protein family members and metabolic enzymes). These results suggest that the protein-level responses to temperature are highly stage-specific, and that the first-feeding stage exhibits a more extensive response.

KEGG enrichment analysis revealed that DEPs at the incubation stage were mainly enriched in glycine, serine and threonine metabolism, 2-oxocarboxylic acid metabolism, and alanine, aspartate and glutamate metabolism pathways (Figure 1c). At 16 °C, vesicle transport proteins (GOSR1, t-SNARE) maintained relatively high expression levels. At 19 °C, amino acid metabolic enzymes such as BHMT, SPT, and AGXT were significantly downregulated, while energy metabolism enzymes, including GK and ACSL/ACSL3, exhibited marked expression fluctuations. Under 22 °C heat stress, transport and antioxidant proteins such as TF, TF2, and ApoA1 were significantly upregulated, whereas the expression of key embryonic developmental enzymes, including AGXT2 and PDXP, decreased (Figure 1c).

DEPs at the first-feeding stage were mainly enriched in the citrate cycle, linoleic acid metabolism, and ferroptosis pathways (Figure 1d). At 16 °C, cell structural proteins such as COL1A1, COL1A2, and FC-NC1 were highly expressed, whereas the overall abundance of digestive enzymes was relatively low. At 19 °C, digestive enzymes (Pepsin A, GAST, trypsin) and lipid metabolism enzymes (FADS2, TGL) were significantly upregulated. At 22 °C, immune- and stress-related proteins such as C3, TF, and CP showed elevated expression, while the expression of structural proteins including COL1A1 was markedly suppressed (Figure 1d).

### 3.3. Metabolomic Differential Response Characteristics

PCA of the metabolomic data similarly showed that the incubation and first feeding-stage samples were clearly separated along PC1, forming two major clusters. Within the first-feeding stage, the 16 °C group was further distinctly separated from the 19 °C and 22 °C groups (Figure 2a). These results further confirm that developmental stage is the dominant factor driving metabolomic variation, and that the first-feeding stage exhibits a stronger response to high temperature. A total of 1292 significantly differentially accumulated metabolites were identified (Table S3), including 593 at the incubation stage and 966 at the first-feeding stage (Figure 2b, Table S4), with 267 metabolites common to both stages (mainly amino acids, nucleotides, and energy metabolism-related molecules), indicating a certain degree of metabolic conservatism between the two stages.

Differential metabolites at the incubation stage were mainly enriched in the biosynthesis of amino acids, fat digestion and absorption, and protein digestion and absorption pathways (Figure 2c). At 16 °C, various free amino acids, including proline, phenylalanine, lysine, tyrosine, histidine, and serine, as well as S-adenosylmethionine, were abundant. At 19 °C, the levels of most amino acids were significantly downregulated, accompanied by the accumulation of organic acids such as pyruvic acid and propionic acid. At 22 °C, acetic acid and propionic acid continued to rise, and the levels of lipid metabolites such as lysophosphatidic acid (18:3) (LPA(18:3)) increased substantially (Figure 2c).

Differential metabolites at the first-feeding stage were mainly enriched in pathways in cancer, purine metabolism, and nucleotide metabolism (Figure 2d). At 16 °C, ADP and valine were abundant, whereas lipids such as lysophosphatidic acid (LPA) and glyceryl arachidonate were relatively low. At 19 °C, LPA, glycerides, thymine, and glycine were markedly enriched, while ADP content decreased. At 22 °C, ADP and valine continued to decline, and β-alanine content increased significantly (Figure 2d).

### 3.4. Integrated Proteomic and Metabolomic Analysis

To further elucidate the molecular mechanisms underlying heat-induced inhibition of embryonic development, KEGG pathway co-enrichment analysis and protein–metabolite correlation analysis were performed for the two developmental stages under the 22 °C treatment. At the incubation stage, the co-enriched pathways of the two omics mainly included fatty acid biosynthesis, pantothenate and CoA biosynthesis, and alanine, aspartate and glutamate metabolism (Figure 3a). Correlation analysis revealed that metabolites such as acetyl-L-carnitine exhibited very strong negative correlations (r < −0.90) with lipid and amino acid metabolic proteins including ACSL3 and AGXT2. In addition, 2,4-dihydroxypteridine and bisindolylmaleimide also showed very strong negative correlations with development-related structural proteins such as SNAP23 (Figure 3b, Table S5).

At the first-feeding stage, the co-enriched pathways shifted to polyunsaturated fatty acid metabolism, ferroptosis, glutathione metabolism, and lysosome pathway (Figure 4a). Correlation analysis showed that fraxinol and dihydrophenazine-1-carboxylic acid exhibited strong positive correlations with the structural protein COL1A1, but strong negative correlations with numerous metabolic enzymes (e.g., SMS, APT1, MAN2B1). Acetylcarnitine was positively correlated with lipid metabolism-related proteins (PLB1) but negatively correlated with ribosomal proteins (EF1A) (Figure 4b, Table S6). Taken together, these results indicate that under heat stress, the incubation stage is characterized predominantly by disrupted lipid and amino acid metabolism, whereas the first-feeding stage shifts to networks associated with oxidative damage and ferroptosis.

## 4. Discussion

Recent field monitoring showed that the water temperature at the spawning ground downstream of the Gezhouba Dam was still as high as 23.1 °C in October [10]. Under the combined effects of intensifying global climate change and reservoir-induced thermal lag, autumn water temperatures at the spawning ground are projected to continue rising, further threatening the natural early development of Chinese sturgeon. In this study, three constant-temperature treatments (16 °C, 19 °C, and 22 °C) were established according to natural water-temperature conditions, and phenotypic, proteomic, and metabolomic data were integrated to systematically compare the responses to heat stress between the embryonic incubation stage (endogenous nutrition) and the larval first-feeding stage (exogenous feeding).

Phenotypically, the 16 °C treatment was optimal for fertilization, hatching, and larval survival. The 19 °C treatment showed sharply reduced hatching but the highest larval specific growth rate. At 22 °C, hatching and larval survival decreased relative to 16 °C. These results are consistent with previous findings on the critical water temperatures for Chinese sturgeon embryonic development [20], and further indicate a clear separation between the optimal temperatures for the embryonic and larval stages [21]. The sharp declines in hatching rate and survival rate observed under 22 °C in this study indicate that the 1–3 °C temperature increase caused by the thermal lag effect has already exceeded the stage-specific tolerance threshold of early Chinese sturgeon development, posing a substantial threat to natural reproduction success. Notably, although 22 °C significantly suppressed embryo and larval survival, it shortened the hatching time from 170 h at 16 °C to 84 h [22]. This phenomenon suggests that high temperature, while accelerating the processes of cell division and differentiation, may impair developmental precision and integrity, ultimately manifesting as a drastic reduction in the hatching rate [23].

Integrative proteomic and metabolomic analyses revealed that the response to heat stress during the incubation stage (endogenous nutrition stage) was characterized predominantly by “metabolic inhibition”. Analysis of metabolite profiles showed that at 16 °C, various free amino acids (proline, phenylalanine, lysine, tyrosine, histidine, and serine) and S-adenosylmethionine were abundant, indicating that under optimal temperature, embryos can orderly mobilize and utilize endogenous nutrients. As the temperature increased to 19 °C, the levels of most amino acids were significantly downregulated, accompanied by the initial accumulation of organic acids such as pyruvic acid and propionic acid. At 22 °C, acetic acid and propionic acid continued to rise, and lipid metabolites such as lysophosphatidic acid (18:3) increased substantially. This metabolic trajectory reflects that elevated temperature disrupts the ordered catabolism of endogenous nutrients, leading to the accumulation of incompletely oxidized intermediates and obstruction of energy metabolism pathways. Embryonic development is a sustained energy-consuming process, with the oxygen consumption rate increasing progressively, especially during the stages of heart formation and beating [24]. The accumulation of organic acids under high temperature implies a reduced efficiency of the tricarboxylic acid cycle, preventing embryos from effectively producing ATP through the complete oxidation of amino acids and fatty acids, thereby trapping them in a vicious cycle of “metabolic inhibition–energy deficiency” [5,25,26].

At the protein level, vesicle transport proteins (GOSR1, t-SNARE) maintained high expression at 16 °C, indicating normal functioning of endomembrane trafficking and secretion under optimal temperature [27]. Under heat stress at 22 °C, transport and antioxidant proteins (TF, TF2, ApoA1) were significantly upregulated, whereas the expression of key embryonic developmental enzymes, AGXT2 and PDXP, was markedly downregulated. AGXT2 (alanine–glyoxylate aminotransferase 2) is involved in amino acid metabolism and glyoxylate detoxification, and PDXP (pyridoxal phosphatase) regulates vitamin B6 metabolism; the downregulation of these two enzymes may further exacerbate the disruption of amino acid metabolism and the accumulation of organic acids [28,29]. Furthermore, the ribosome pathway was significantly enriched and contained numerous DEPs during embryonic development, underscoring its particular importance at the endogenous-nutrition stage, when ribosomes serve as the core machinery for protein biosynthesis [30]. Dysregulated expression of ribosome-related proteins suggests that heat stress disrupts fundamental protein-synthesis processes. Furthermore, correlation analysis between differentially expressed proteins and metabolites at the incubation stage revealed a very strong negative correlation (r < −0.90) between acetyl-L-carnitine and lipid/amino acid metabolic proteins (ACSL3, AGXT2), as well as between 2,4-dihydroxypteridine and bisindolylmaleimide and the development-related structural protein SNAP23. These strong correlations indicate a tight coupling between aberrant metabolite accumulation and the suppressed expression of key functional proteins under high temperature, jointly forming the molecular network of “metabolic inhibition” [31].

In contrast to the incubation stage, the heat stress response during the first-feeding stage (exogenous nutrition stage) was dominated by “oxidative damage–ferroptosis”. The shift reflects distinct metabolic modes, with embryos relying on endogenous yolk and relatively anaerobic metabolism, whereas first-feeding larvae depend on exogenous feeding and aerobic metabolism. The enhancement of aerobic metabolism inevitably leads to increased production of reactive oxygen species (ROS) in the mitochondrial electron transport chain [32]. Under optimal temperatures, larvae can effectively scavenge ROS through antioxidant pathways such as glutathione metabolism; however, under heat stress at 22 °C, the rate of ROS generation exceeds the scavenging capacity of the antioxidant system, thereby triggering oxidative stress [33,34,35]. In this study, the significant enrichment of the ferroptosis pathway and polyunsaturated fatty acid metabolism pathway in the 22 °C treatment group indicates that excessive ROS attack phospholipids rich in polyunsaturated fatty acids in cell membranes, initiating a chain reaction of lipid peroxidation and ultimately activating ferroptosis, a form of programmed cell death dependent on iron and lipid peroxidation [36,37]. Previous studies in model organisms such as zebrafish have demonstrated that heat stress can induce oocyte and embryonic damage through lipid peroxidation [38,39,40]; this study provides the first proteomic and metabolomic evidence of heat stress-induced ferroptosis in an endangered sturgeon species.

At the protein level, cell structural proteins (COL1A1, COL1A2, FC-NC1) were highly expressed at 16 °C, while the overall abundance of digestive enzymes was relatively low, suggesting that larvae at this temperature primarily maintained basal structural integrity rather than engaging in active feeding and growth [41]. At 19 °C, digestive enzymes (pepsin A, GAST, trypsin) and lipid metabolic enzymes (FADS2, TGL) were significantly upregulated, closely matching the peak in larval specific growth rate and indicating that 19 °C is favorable for the initiation of exogenous feeding and rapid growth [42]. In contrast, at 22 °C, immune and stress-related proteins (C3, TF, CP) showed elevated expression, whereas the expression of the structural protein COL1A1 was markedly suppressed. This shift in protein expression profiles from “structural inhibition” to “stress activation” reveals the adaptive cost incurred by larvae under heat stress, whereby limited energy resources are diverted from growth and structural maintenance toward stress responses [43,44].

The metabolomic data also supported the shift toward the “oxidative damage–ferroptosis” pattern. The 16 °C group was characterized by abundant ADP and valine, along with relatively low levels of lipids such as lysophosphatidic acid (LPA) and glyceryl arachidonate. When the temperature increased to 19 °C, LPA, glycerides, thymine, and glycine were markedly enriched, while ADP content declined—a metabolic profile that corroborated the upregulation of digestive enzymes and lipid metabolic enzymes observed in the proteomic data, collectively reflecting active lipid synthesis and energy metabolism following the initiation of exogenous feeding [45,46]. At 22 °C, ADP and valine continued to decline, whereas β-alanine content increased significantly. The accumulation of β-alanine may serve as an indicator of intensified nucleotide degradation under oxidative stress [47], further supporting the conclusion that high temperature leads to energy depletion and oxidative damage.

A comprehensive comparison of the molecular responses between the incubation and first-feeding stages reveals completely distinct core vulnerable pathways, with the incubation stage dominated by amino acid and fatty acid metabolic disruption, organic acid accumulation, and energy deficiency and the first-feeding stage characterized by polyunsaturated fatty acid peroxidation, ferroptosis activation, and lysosomal pathway abnormalities. Only a few heat shock proteins (HSPs) and basic metabolites were commonly upregulated in both stages, suggesting that protein misfolding represents a universal threat under heat stress [48], yet the substantive damaging pathways are entirely determined by the metabolic and physiological state of each developmental stage. The fundamental cause of this divergence lies in the critical transition from endogenous nutrition to exogenous feeding, with the latter accompanied by a burst of mitochondrial ROS production [49], rendering oxidative damage the predominant lethal factor.

## 5. Conclusions

Our results demonstrate that thermal lag-related warming exerts distinct deleterious effects on Chinese sturgeon embryos and first-feeding larvae, pointing to dual physiological threats to early-life survival. Divergent core injury pathways correspond to the metabolic transition from endogenous yolk nutrition to exogenous feeding. For conservation, our results highlight that 22 °C exceeds the upper thermal limit for natural reproduction of Chinese sturgeon and severely compromises its early development. The limitations of this laboratory study include the limited number of parental broodfish and the constant-temperature experimental design. Further experiments using more independent parental families are required. In addition, further work should validate these findings under fluctuating thermal conditions and conduct functional validation of key pathways such as ferroptosis.

## Figures and Tables

**Figure 1:**
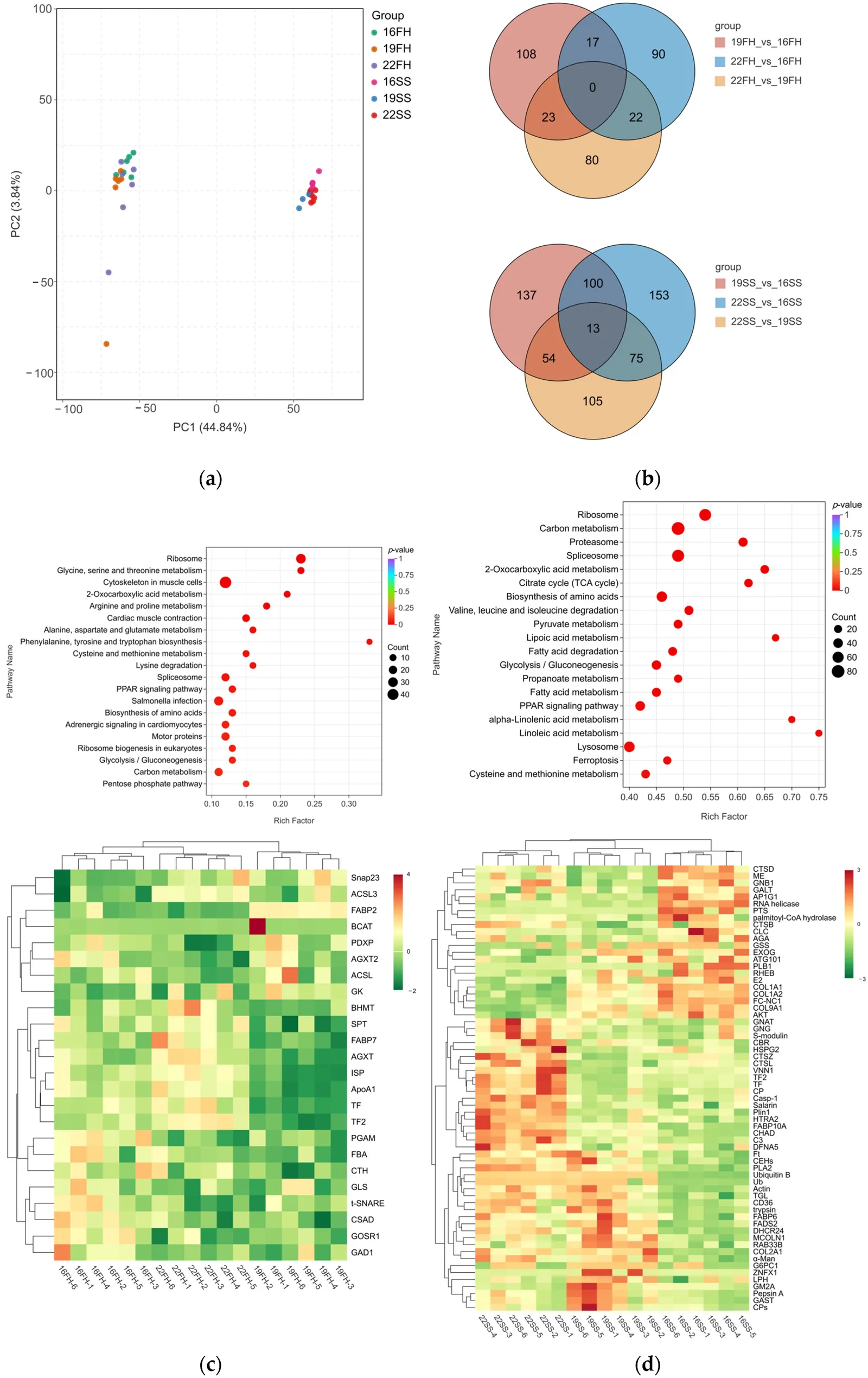
Proteomic profiling of samples at the fertilized-hatching stage (FH) and first feeding stage (SS) under different temperatures. (**a**) Two-dimensional PCA plot; (**b**) Venn diagram of differentially expressed proteins (DEPs) among temperature subgroups at the FH and SS stage; (**c**) bubble plot of KEGG pathway enrichment for DEPs from FH-stage comparisons, and heatmap showing expression patterns of representative DEPs; (**d**) bubble plot of KEGG pathway enrichment for DEPs from SS-stage comparisons, and heatmap showing expression patterns of representative DEPs.

**Figure 2:**
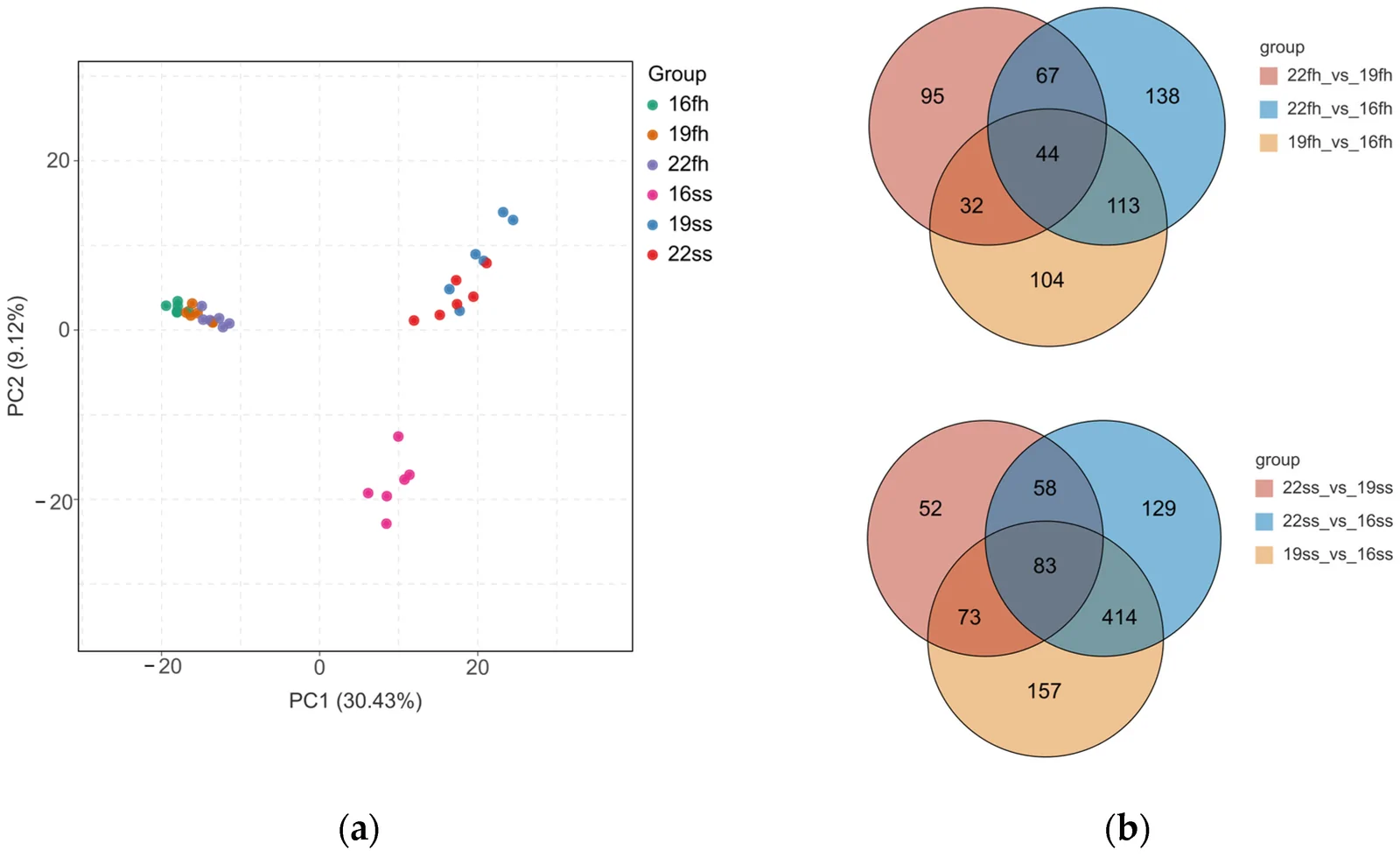

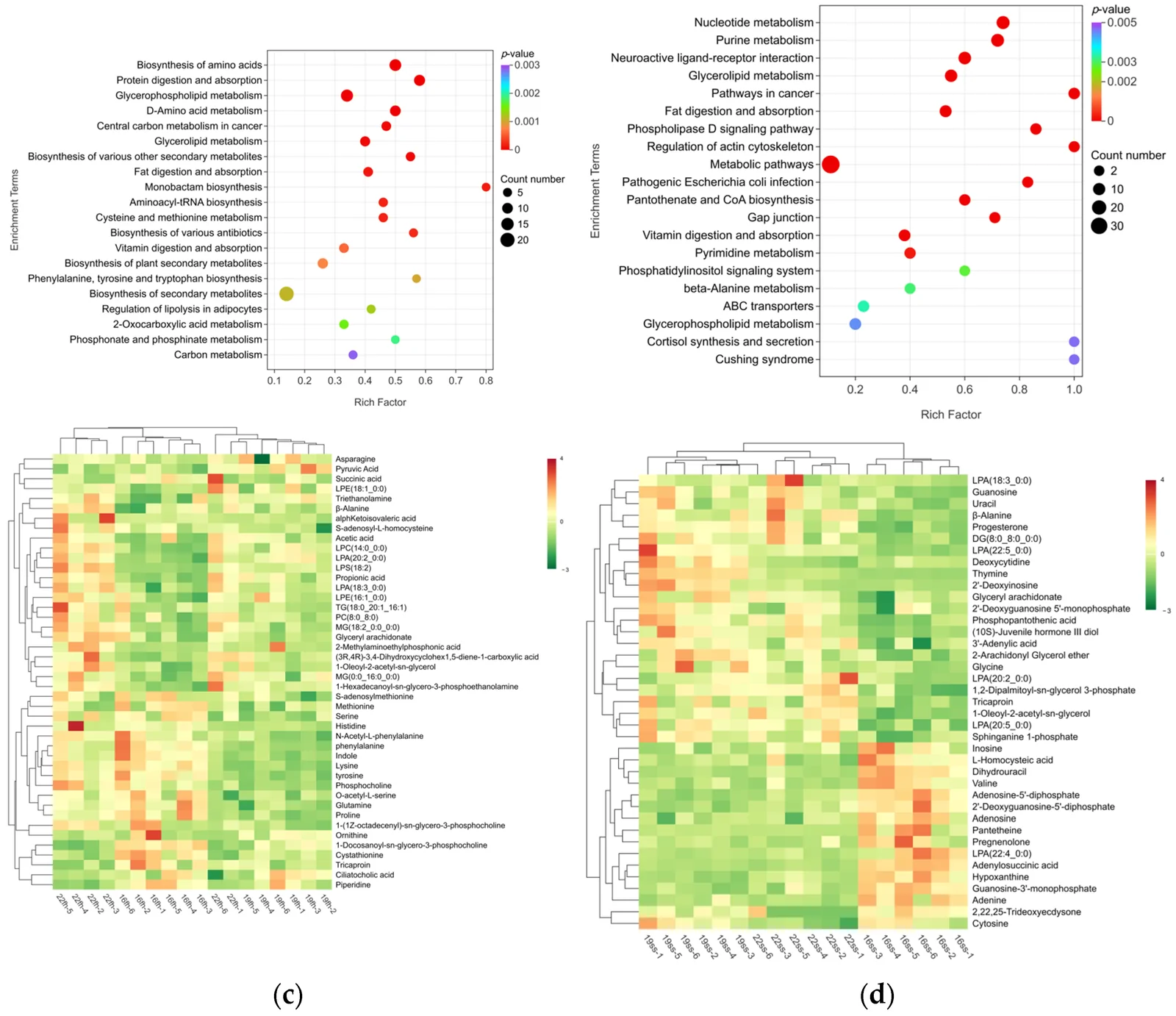
Metabolomic profiling of samples at the fertilized-hatching stage (fh) and first-feeding stage (ss) under different temperatures. (**a**) Two-dimensional PCA plot; (**b**) Venn diagram of differentially abundant metabolites (DAMs) among temperature subgroups at the fh and ss stage; (**c**) bubble plot of metabolite set enrichment analysis (MSEA) for DAMs from fh-stage comparisons, and heatmap showing expression patterns of representative DAMs; (**d**) bubble plot of MSEA for DAMs from ss-stage comparisons, and heatmap showing expression patterns of representative DAMs.

**Figure 3:**
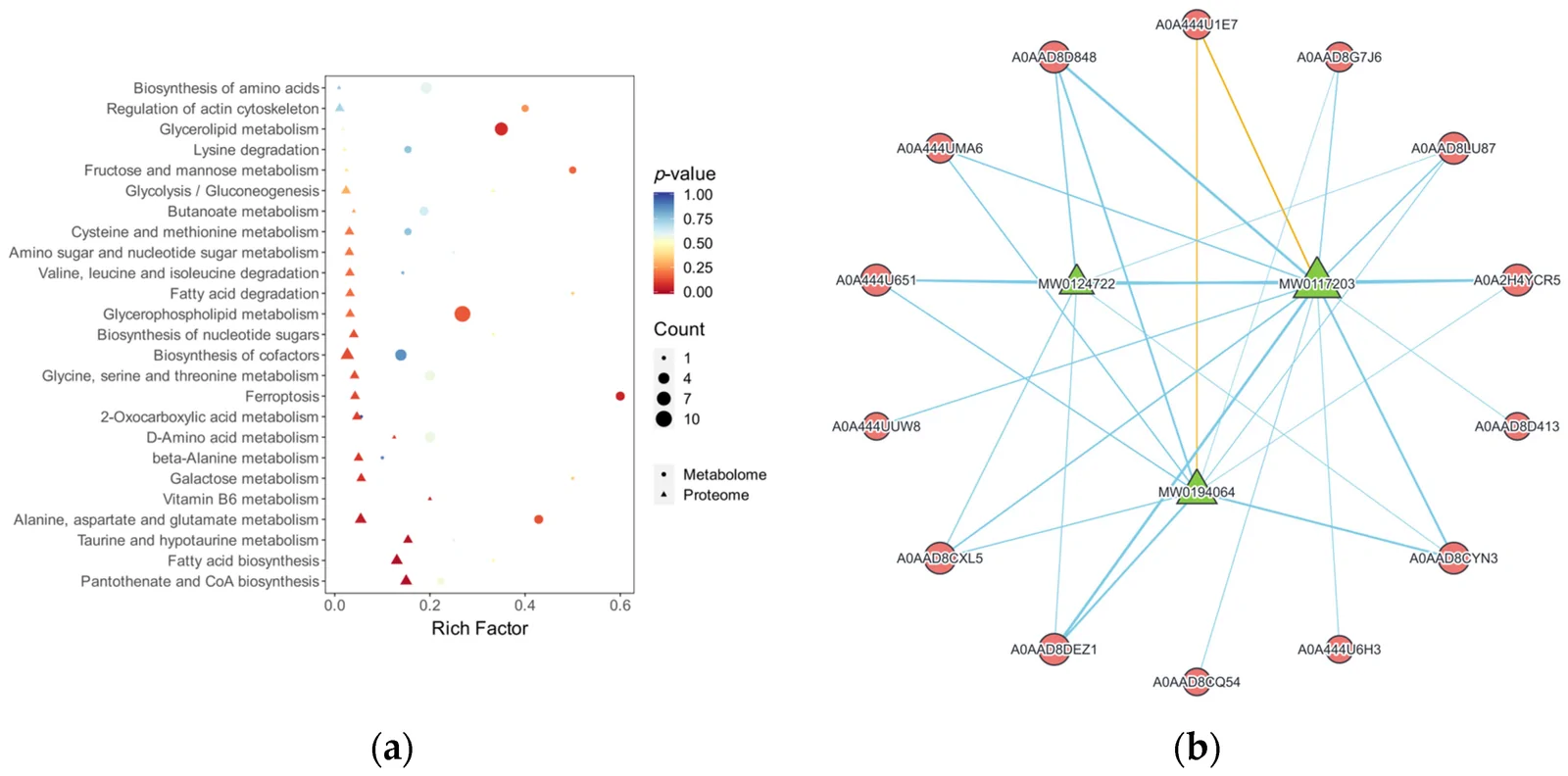
Integrated proteome and metabolome analysis of samples at the fertilized-hatching stage. (**a**) Comparative bubble plot of enriched KEGG pathways based on differentially expressed proteins (DEPs) and differentially abundant metabolites (DAMs); (**b**) correlation network of key DEPs (circular nodes) and DAMs (triangular nodes). Orange and cyan lines stand for positive and negative correlations, respectively.

**Figure 4:**
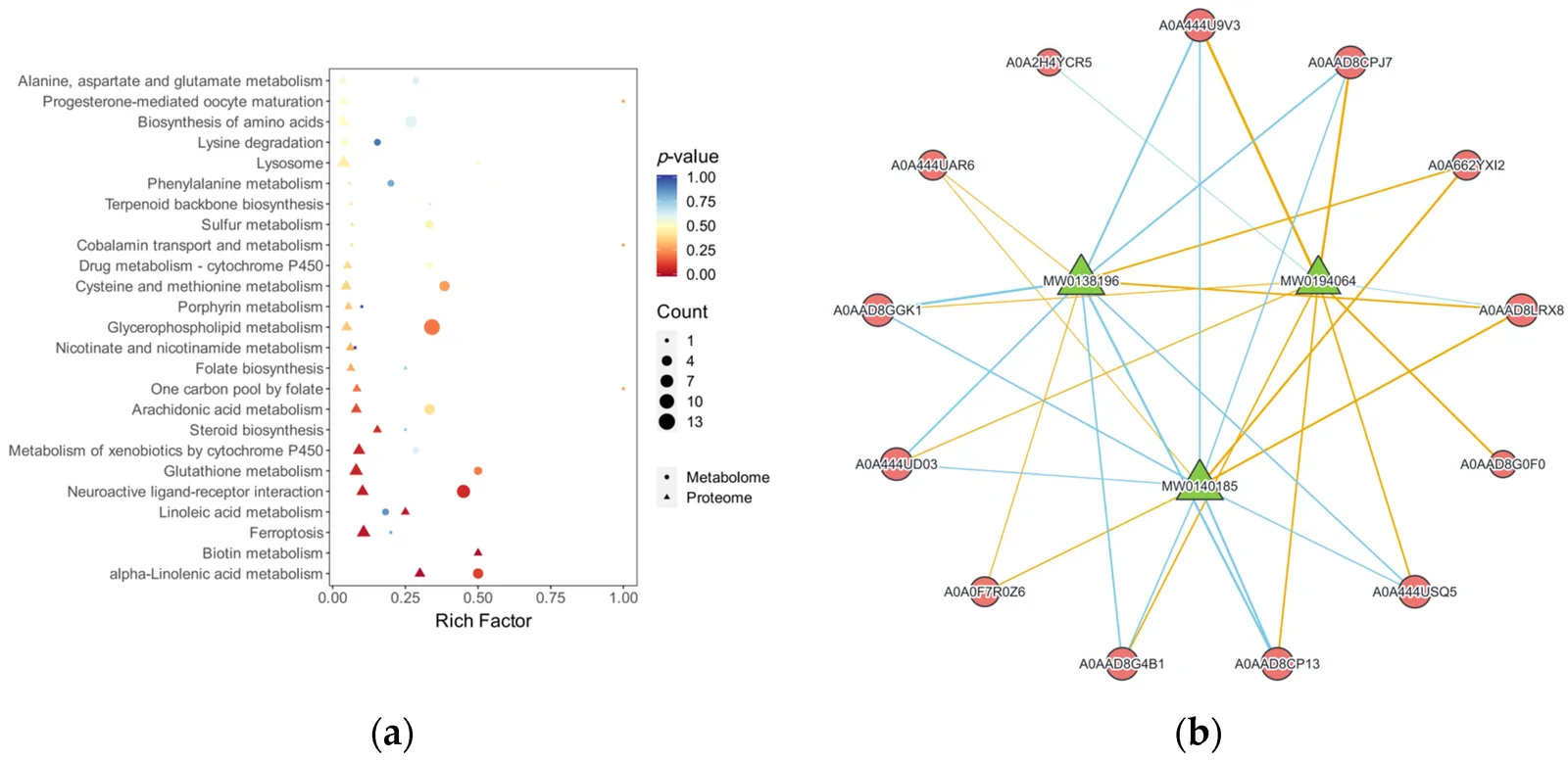
Integrated proteome and metabolome analysis of samples at the first-feeding stage. (**a**) Comparative bubble plot of enriched KEGG pathways based on differentially expressed proteins (DEPs) and differentially abundant metabolites (DAMs); (**b**) correlation network of key DEPs (circular nodes) and DAMs (triangular nodes). Orange and cyan lines stand for positive and negative correlations, respectively.

**Table 1:** Phenotypic parameters of embryos and larvae reared at three temperatures (16 °C, 19 °C, and 22 °C).

Temperature Treatments	Incubation Stage			Exogenous Feeding Stage	
	Fertilization Rate (%)	Hatching Duration (h)	Hatching Rate (%)	Survival Rate (%)	Specific Growth Rate (%/d)
16 °C	97.5 ± 0.6 ^a^	170	64.6 ± 0.8 ^a^	87.6 ± 2.3 ^a^	3.71 ± 0.68 ^b^
19 °C	96.5 ± 0.9 ^a^	104	23.1 ± 3.4 ^b^	78.6 ± 3.3 ^b^	6.79 ± 0.93 ^a^
22 °C	94.6 ± 0.7 ^b^	84	15.0 ± 4.9 ^b^	75.4 ± 2.7 ^b^	5.95 ± 2.20 ^a^
r	−0.984	−0.955	−0.932	−0.964	0.703

Note: Values are presented as mean ± SD. Different superscript letters within the same column indicate significant differences among temperature treatments (*p* < 0.05). r Pearson correlation coefficients between temperature and each phenotypic parameter.

## Data Availability

The original contributions presented in this study are included in the article. Further inquiries for raw data can be directed to the corresponding authors.

## Supplementary Materials

The following supporting information can be downloaded at: https://www.mdpi.com/article/10.3390/biology15191713/s1, Table S1: List of 931 significantly differentially expressed proteins (DEPs) under 16/19/22 °C at the fertilized-hatching stage (FH) and first-feeding stage (SS); Table S2: List of 340 differentially expressed proteins (DEPs) at the fertilized-hatching stage (FH) and 637 DEPs at the first-feeding stage (SS); Table S3: List of 1292 significantly differentially abundant metabolites (DAMs) under 16/19/22 °C at the fertilized-hatching stage (fh) and first-feeding stage (ss); Table S4: List of 593 differentially abundant metabolites (DAMs) at the fertilized-hatching stage (fh) and 966 DAMs at the first-feeding stage (ss); Table S5: Correlation analysis between representative DEPs and DAMs at the fertilized-hatching stage. Note: absolute Pearson correlation coefficient > 0.9 and *p* < 0.05; Table S6: Correlation analysis between representative DEPs and DAMs at the first-feeding stage. Note: absolute Pearson correlation coefficient > 0.9 and *p* < 0.05.

## Abbreviations

The following abbreviations are used in this manuscript:

ACSL3	Acyl-CoA synthetase long-chain family member 3
ADP	Adenosine diphosphate
AGXT2	Alanine-glyoxylate aminotransferase 2
ANOVA	Analysis of variance
ApoA1	Apolipoprotein A1
APT1	Acyl-protein thioesterase 1
ATP	Adenosine triphosphate
BCA	Bicinchoninic acid
BHMT	Betaine-homocysteine S-methyltransferase
C3	Complement component 3
COL1A1	Collagen type I alpha 1 chain
COL1A2	Collagen type I alpha 2 chain
CoA	Coenzyme A
CP	Ceruloplasmin
CR	Critically Endangered
DAMs	Differentially accumulated metabolites
DEPs	Differentially expressed proteins
DIA	Data-independent acquisition
DTT	Dithiothreitol
EDTA	Ethylenediaminetetraacetic acid
EF1A	Elongation factor 1 alpha
FADS2	Fatty acid desaturase 2
FC	Fold change
FDR	False discovery rate
FH/fh	Fertilized-hatching stage
GAST	Gastricsin
GK	Glucokinase
GOSR1	Golgi SNAP receptor complex member 1
HCA	Hierarchical cluster analysis
HCD	High-collision dissociation
HSPs	Heat shock proteins
IUCN	International Union for Conservation of Nature
KEGG	Kyoto Encyclopedia of Genes and Genomes
KNN	K-nearest neighbors
LC-MS	Liquid chromatography–mass spectrometry
LPA	Lysophosphatidic acid
MAN2B1	Mannosidase alpha class 2B member 1
MaxLFQ	Max label-free quantification
MBR	Match-between-runs
MSEA	Metabolite set enrichment analysis
OPLS-DA	Orthogonal partial least-squares-discriminant analysis
PCA	Principal component analysis
PDXP	Pyridoxal phosphatase
PLB1	Phospholipase B1
PMSF	Phenylmethylsulfonyl fluoride
QC	Quality control
QE	Q Exactive
ROS	Reactive oxygen species
rpm	revolutions per minute
SD	Standard deviation
SMS	Sphingomyelin synthase
SNAP23	Synaptosome-associated protein 23
SPT	Serine palmitoyltransferase
SS/ss	First-feeding stage
SVR	Support-vector regression
TF	Transferrin
TGL	Triacylglycerol lipase
t-SNARE	Target soluble N-ethylmaleimide-sensitive factor attachment protein receptor
UHPLC	Ultra-high-performance liquid chromatography
VIP	Variable importance in projection
